# Notes from Private Practice

**Published:** 1878-04

**Authors:** 


### NOTES FROM PRIVATE PRACTICE.

*Chronic Anæmia, Associated with Pregnancy.*

Mrs. B——, aged 26 years, married, and of healthy parentage, came to my office for treatment July 7th, 1877. She was then in the eighth month of pregnancy, was very anæmic, and complained of great debility, irregular action of the heart, pain in the back, epigastrium and right side, loss of appetite, etc. She stated that she had been confined two years ago and had then had a severe attack of child-bed fever, never having been well since. She was somewhat emaciated, and presented a cachectic appearance. The integument of the face, hands, and a part of the body, were of a bronzed hue. The tongue, gums, lips, and mucous membrane of the mouth were bloodless ; the tongue large and flabby, and its mucous membrane displayed five or six ashy looking ulcers. Pulse, 120, weak and irregular. Respirations 20 to 25, and greatly embarrassed. The action of the heart was tumultuous, and upon auscultation at the base of the heart, a loud systolic bruit could be heard over the aortic and pulmonary valves.

The urine was of an amber color, and acid reaction ; deposits when cool a sediment composed of urates, phosphates, and uric acid ; specific gravity 1.010, no albumen or sugar.

*Diagnosis : Chronic Progressive Anæmia.*—She was ordered iron, quinine, strychnia and phosphorus, with a highly nutritious diet.

July 11th.—Some improvement ; appetite better, and is not quite so weak ; continued same treatment.

July 13th.—Was called to see her, and found her suffering from an attack of remittent fever, attended with continuous nausea and vomiting, and neuralgic pains in almost every part of the body; great dyspnœa, throbbing of the carotids, and the impulse of the heart could be felt over very nearly the whole of the anterior portion of the chest. The pulse was so frequent and irregular that its rate could not be definitely noted, but ranged from 120 to 150, and was very feeble; temperature 99½° F.; inability to assume the upright position without danger of fainting. I prescribed digitalis, belladonna, morphia, and potassic bromide, and moved the bowels with a mild laxative, giving quinine every two hours. To relieve the nausea and vomiting, mustard was applied over the stomach; she also had bismuth, lime-water, and sweet milk every hour; extract of beef also to be given every hour or two.

July 16th.—Some improvement.

July 17th.—The fever was relieved.

July 18th.—Patient doing well; ordered that the iron, quinine, strychnia, and phosphoric acid be given again as before, it having been discontinued during the attack of fever. She continued to improve from that time, and on the night of the 10th of August I was summoned to attend her in labor.

When I arrived at the house and saw her, I was astonished at the great improvement she had made; the pallor had left the mucous surfaces, the heart was now acting normally, and she expressed herself as being well. Her labor pains were feeble, and upon vaginal examination, I found that the os had not dilated, neither was it affected by the pains. Being satisfied that labor had not set in, I gave her an opiate and left.

On the 25th I was again called to attend her, and found her in active labor, the os dilating. Labor progressed well for one or two hours and then ceased, the os refusing to dilate, and becoming rigid and very sensitive. Under the use of hot baths, chloral, morphia, and rupturing the membranes, it gave way, and she was delivered of a fully matured fœtus in a state of decomposition. The patient made a rapid recovery and is now in the best of health. In conclusion, I desire to call attention to a few points in this case: 1st. To the rapid recovery of this patient under such unfavorable circumstances, viz., pregnancy existing as a complication. 2d. To the efficiency of this line of treatment, viz., iron, quinia, strychnia, and phosphoric acid, with good diet. 3d. To the length of time the fœtus remained in utero after its maturity and death; for, according to her reckoning, labor should have occurred on the 10th day of August, at the time she had spurious pains. The patient says that she never felt any movements of the child after that date. Then we must conclude she carried a dead fœtus from the 10th to the 25th of August.

J. M. MATHES, M. D.

CARLISLE, Ind., Dec. 29th, 1877.

### *Recovery from Poisoning by six drachms of Chloroform taken in the Stomach.*

I was recently called at 10:30 a. m., to see a young lady who had just been found in an unconscious condition, presumably from the effects of an overdose of chloroform.

From what I learned of the family I think that the patient had taken the poison half or three quarters of an hour before I saw her; I found her thorougly anæsthetized, respiration nearly normal and pulse eighty per minute.

The patient, who had already vomited profusely, vomited again shortly after I entered the room, so that there seemed no need of emetics or the stomach pump.

From the odor of bottles found in the apartment, I suspected that the patient had taken hydrate of chloral, but there was no means of ascertaining the quantity as she had carefully removed the label from the bottle in which the poison had been procured.

I sent at once for carbonate of ammonia; but when it arrived the patient was unable to swallow, the respiratory efforts were becoming inefficient and the pulse was gradually failing.

The patient was given frequent inhalations of ammonia, and the twentieth of a grain of sulphate of strychnia by hypodermic injection; another dose of one-fortieth of a grain of sulphate of strychnia was given about three quarters of an hour later. Finding the action of the heart and respiratory muscles steadily failing, I dispatched a messenger for an electric battery, but before his return, and while I was alone with the patient, the respiratory efforts ceased so that life was only sustained by artificial respiration. In this emergency I sent for Drs. E. Ingals and Norman Bridge; both soon came in and assisted me for the next hour and a half.

Artificial respiration became necessary about two hours after the poison had been taken. Electricity was applied about half an hour later, one pole to the nape of neck and the other to the pit of the stomach and various portions of the chest. The pulse by this time had fallen to fifty-two per minute and the respiration was wholly artificial for some time.

From twelve o'clock until half past one, artificial respiration was maintained, and during the greater portion of the time the battery was in frequent use.

During a considerable part of this time if artificial respiration was suspended there would follow a few faint respiratory efforts which would then cease, so that had the patient been left for five minutes death would surely have been the result.

About an hour after the first dose of strychnia, considerable rigidity of the muscle of the arms occurred and continued for fifteen or twenty minutes.

Perfect anæsthesia was maintained for three hours, when a slight movement of the eye, upon touching the conjunctiva, indicated returning consciousness. Half an hour later we noticed the first voluntary movement and at three p. m., about five hours after the drug had been taken, the patient awoke. Respiration had then been regular for some time; a half hour later I left the patient, conscious and able to swallow the strong coffee which had been prepared for her.

I called again in a couple of hours and found her comfortable excepting for frequent attacks of nausea and vomiting. She informed me that she had taken twenty cents worth of chloroform, which from her description was a trifle more than six drahms.

The patient refused to give the name of the person who sold her the chloroform, and probably it would be of no use to find out, for people who wish to take poison will always find some druggist willing to sell it to them; and if it be true, as my friend Dr. Brower is disposed to believe, that nine tenths of those who attempt suicide are insane, possibly they who encourage these unfortunates in their efforts at self destruction, are as kind as the physicians who attempt to thwart them.

E. FLETCHER INGALS, M. D.

188 CLARK STREET, CHICAGO.

*Enormous Dilation of Sigmoid Flexure, with History of Case.*

I was called January 31st, 10 a. m., to see J. B. Farmer, aged 51 years. He had been subject to occasional attacks of "bilious colic" for nearly ten years, during which he vomited frequently and severely, and suffered from severe pain in the bowels. He had been troubled with constipation, especially preceding these attacks. He had also not felt well for nearly a week, and the day previous, began having severe paroxysmal pain in the bowels, accompanied by nausea and vomiting, which continued during the night and were still present. The skin was about normal; tongue dry, white fur in center; pulse 70, soft; temperature normal; appetite lost; bowels constipated; abdomen slightly tympanitic and somewhat tender; no tumor could be felt; the material ejected by vomiting consisted of bile, mucus and fluids taken into the stomach. I administered hypodermically morphia sulph. gr. $\frac{3}{8}$, atropia sulph. gr. 1-60; ordered a large hot poultice applied to the abdomen continuously, and small bits of ice given ad libitum; also, bismuth sub. nit. gr. vi. every four hours. I also left a powder containing ten grains of calomel, to be given at night. I called early next morning; the calomel had not operated; there had been no pain nor vomiting since last visit. Pulse 68, temp. 98°, resp. 20. I administered one tablespoonful of rochelle salts, and after waiting several hours, used an enema which was passed at once without any fæcal matter. I left another dose of salts to be given at noon if the bowels had not moved.

I was called again at 2 p. m. in haste, to find my patient suffering great pain in the hypogastric region, and vomiting occasionally. His bowels had not moved; several enemata had been given; pulse 72, temp. 99°. I at once gave up any further effort to move the bowels, and administered a hypodermic injection as before. Patient soon became quiet, and rested well for several hours. The poultice was continued, and fluid nourishment given freely. At 8 o'clock p. m. he was suffering some pain in bowels; no vomiting; pulse 70, and had passed some mucus from the bowels two or three times with considerable tenesmus. At 10 o'clock p. m. the pain very severe; abdomen more tympanitic. I administered another hypodermic injection, which gave temporary relief.

The patient frequently tried to eject gas from the stomach by belching. The swelling of the abdomen kept increasing until midnight, becoming finally enormous, and causing great distress. Pulse 78, temp. 99°, resp. 36, short and labored. I administered various carminatives, but without relief; ordered turpentine stupes and used a rectal gum elastic tube, hoping to relieve the colon of gas, but could not pass it beyond the sigmoid flexure. The patient begged to have the "wind pumped out of his stomach." I passed a No. 10 catheter into the stomach, when quite a quantity of gas escaped. The abdomen was so tense that it seemed as if the gut must rupture. A distinct ridge revealed (as I thought) the outline of the distended ascending colon. I now left him, to get a trocar, intending to tap the colon. When I returned two hours later (3 o'clock a. m.), his suffering was most intense; pulse 140, temp. 102°, resp. 40. His wife would not permit the trocar to be used. I therefore administered injections per rectum of a decoction of tobacco, which seemed to give temporary relief.

I kept him quiet with hypodermic injections until Doctor S. M. Hamilton, of Monmouth, who had been called in consultation, agreed with me as to the necessity of tapping, and very skillfully operated, perforating, as we supposed, the ascending colon, and giving vent to a large quantity of offensive gas. This gave great relief. The respiration fell to 30, pulse, 132; abdomen quite tender.

He still passed mucus occasionally with tenesmus. I continued the opiates and concentrated fluid nourishment with whisky, and yielding to the wishes of the friends, injections were administered occasionally of a solution of ox gall. His wife thought that "if he could only have a passage from the bowels he would get well."

At eight o'clock p. m., the patient was delirious; no pain; pulse 140, small; abdomen distending rapidly. At 10 p. m., delirium continued, the abdomen was very tense; respiration 40. At 11 o'clock I tapped the bowels again about half an inch above first puncture, permitting the escape of a still larger quantity of gas.

The respiration fell to 24; pulse 144, and thready. He rested quietly until about 3:30 o'clock a. m., when he began to sink, and died at about half past four.

Autopsy ten hours after death. Rigor mortis marked; on opening the abdominal cavity, I cut down upon what proved to be an immense sac-like dilatation of the sigmoid flexure, which entirely covered the anterior surface of the bowels, and which we had punctured on the right side, instead of the ascending colon; it extended as high as the cruciform cartilage, and was perfectly black from congestion. Further examination revealed extensive enteritis, and general peritonitis. The sac was empty, its walls thick and muscular, and it would hold at least a gallon. No fæcal accumulation in any part of the bowels; no apparent contraction below the sac; no morbid deposit in walls of rectum. The liver and spleen were congested and somewhat softened; other organs healthy.

*Query.* At what time did general inflammation begin? Did the purgatives administered light up, or only aggravate an already exisiting entero-peritonitis? I did not diagnosticate the above until within 24 hours of death. Did it exist prior to that time, with a pulse rate of 70, and temperature of 98½°? Will some one please answer?

H. L. HARRINGTON, M. D.

WARREN CO., ILLS.

*The Value of Electricity in Paralysis.*

Dr. Brown-Séquard, having recently lectured in our city, the following case may be more than usually interesting. The chief object in reporting it is, however, for the purpose of calling the profession to the importance of the immediate use of electricity in these cases, instead of delaying for several days as recommended by most writers upon the subject.

Let it be remembered that the sooner the function of respiration, for instance, is restored—when stopped by hanging, drowning or other causes of suffocation—and the shorter the duration of its broken continuity, the better will be the chances in both instances for the patient's recovery.

This will also be strikingly observed in the causes which produce anchylosis.

The deduction to be made is, this : *the quicker the nerve cells are aided in the performance of lost function, the greater hope there will be of restoring them to permanent normal activity.* Delay here must therefore be dangerous if anywhere.

What, then, can accomplish this quicker than electricity ?

Case: Mrs. C——, an apparently healthy married lady, American, age 27, seven and one half months pregnant; somewhat subject to insomnia and nervousness ; the mother of three children.

On the 17th of last December, after running a couple of blocks to catch a street car, she was suddenly seized with total paralysis of the right side of the body and face. When seen, two and one-half hours subsequently, she was found in this condition: face contorted and livid; right pupil slightly dilated; forehead a trifle hot ; right arm and hand a little cool; she was entirely speechless and vision obscured; perceptions blunted; hearing obtuse; breathing somewhat stertorous; pulse normal; respiration 12 : heart not examined unfortunately. Ordered cold to the head and warmth to the feet, and applied immediately *with gentle power*, the positive electrode of a Kidder's machine to the base of the cerebellum, the other to the foot, alternately to the hand, retaining the first at the neck, during the whole time of employing the current, about half an hour.

After it was applied in this manner for five or eight minutes partial articulation was possible and the power of mobility partially returned; both became almost normal at the end of one hour. But, owing to so many friends calling, in mistaken kindness to see her, all the former symptoms returned after a cessation of three hours.

May I say that when called an hour after the second attack I had little regret ? Let me state that the therapeutic value of the electricity was so thoroughly proven by the second application acting as promptly and efficaciously as in the first attack, that it seemed a positive demonstration of its value, and left scarcely any room for doubt. The patient was treated, for a few days, with 1-20 grain doses of strychnia and grain doses of quinine. Six weeks after the attacks she had a perfectly natural labor and a moderately healthy child.

For two months previous to the paralysis she had been subject to slight opisthotonos every two or three days; since then these attacks have been much less frequent. On account of the pregnancy, I did not push the strychnia to its full therapeutical action. It seems probable that the dorsal tetanus may pass away upon resuming the strychnia treatment. Much experience has taught the writer the great value of electricity in nerve-cell difficulties, when used promptly and of *moderate* strength.

WM. E. RENNOLDS.

---

SPREAD OF CONTAGIOUS DISEASES.—A contemporary medical periodical has recently called attention to the possibility of transmitting [illegible]ntagious diseases by the medium of books in public libraries[illegible]hich frequently circulate between the homes of the healthy a[illegible]he abodes which are stricken with pestilence.

Another method of extending the contagion, certainly of some of the exanthemata, may be found to be the handling of articles in the shops of dry goods merchants. We recently saw in such an establishment a young girl inspecting some materials for dress, whom we recognized as the inmate of a household, one member of which had variola. Surely the next lady, unprotected by vaccination or previous diseases, who might chance to handle the same goods, would run a terrible risk.

A MEETING of the University of London was held January 15th to consider the new supplemental charter providing for the admission of women to degrees in all the faculties. The question was warmly debated. The vote stood 242 in favor of and 132 against it. The university will ask the government for powers to grant the same degrees to women as it now grants to men.

